# Seismic Behavior of Concrete Columns Reinforced with Weakly Bonded Ultra-High Strength Rebars and Confined by Steel Tubes

**DOI:** 10.3390/ma16216868

**Published:** 2023-10-26

**Authors:** Jing Luo, Shiyu Yuan, Jun Zhao, Yuping Sun

**Affiliations:** 1 Department of Architecture, Graduate School of Engineering, Kobe University, Kobe 657-8501, Japan; 18236911339@163.com (J.L.); yuanshiyu@person.kobe-u.ac.jp (S.Y.); 2 School of Water Conservancy and Civil Engineering, Zhengzhou University, Zhengzhou 450001, China; zhaoj@zzu.edu.cn

**Keywords:** circular columns, WBUHS rebars, bolted steel tubes, resilience, numerical analysis

## Abstract

The usage of weakly bonded ultra-high strength (WBUHS) rebars has emerged as a promising approach to enhance the resilience of concrete components due to their remarkable mechanical properties. To promote the application of WBUHS rebars, this paper presented an investigation on the seismic behavior of circular concrete columns reinforced with squarely arranged WBUHS rebars and externally confined by bolted steel tubes. Eight columns, including two reinforced with normal strength (NS) rebars and six reinforced with WBUHS rebars, were fabricated and tested under reversed cyclic lateral loading. Experimental results showed that the columns reinforced with WBUHS rebars exhibited remarkable drift-hardening capacity up to the drift of at least 5% as well as significantly reduced residual deformation even when subjected to relatively high axial compression with an axial load ratio of 0.33 in comparison to the traditional ductile columns reinforced with NS rebars. Notably, the precast columns reinforced with WBUHS rebars, with an embedment length of 20 times their diameter, behaved nearly identically in terms of resilience as cast-in-place columns. Additionally, a numerical analysis was performed to assess the hysteretic performance, and the analytical results, with consideration for the slippage of WBUHS rebars, were capable of capturing the hysteretic performance of test columns.

## 1. Introduction

As suggested from the recent catastrophic earthquakes, such as the 2008 Wenchuan earthquake, the 2011 Great East Japan earthquake, and the 2023 Turkey–Syria earthquake [1,2,3], code-compliant ductile reinforced concrete (RC) components have played a crucial role in guaranteeing life safety during seismic hazards owing to their excellent deformability and energy-dissipation capacity, whereas the left large residual deformation after earthquakes easily results in substantial economic burden due to business downtime, repair or reconstruction.

The concept of “resilient cities” has garnered increasing attention within seismic design because it puts emphasis on reducing the permanent post-earthquake deformation and swift restoration of essential building functions [4,5]. Numerous endeavors have been undertaken by earthquake engineering researchers to minimize residual deformation and fulfill the requirement for structural repair. In 1993, an early study on the utilization of unbonded prestressing tendon (UPT) was conducted to develop self-centering components with reduced residual deformation [6], and this application of UPT was then further extended by Priestley et al., Zatar et al. and Marriott et al. [7,8,9]. To facilitate construction and avoid the negative impact on seismic performance caused by prestress loss, Ou et al. [10] introduced the utilization of unstressed steel strands as longitudinal reinforcement in RC concrete columns. The results showed that employing unstressed steel strands to replace normal deformed bars was a viable means of effectively controlling residual deformation after unloading the lateral loads. Also, the application of partially debonded reinforcements has been experimentally verified as feasible to obtain the self-centering capacity of concrete columns [11,12,13].

The strengthening and/or repair of earthquake-damaged RC and masonry structures have experienced dramatic growth because of the frequent occurrence of earthquake disasters [14,15]. Initially, fiber-reinforced polymer (FRP) materials were employed for seismic strengthening of RC and masonry structures [16,17], or to address the corrosion of ordinary steel bars [18,19]. Subsequently, FRP materials were processed into straight bars to develop self-centering concrete components due to their high tensile strength and linear elasticity. Mohamed et al. [20,21] conducted an experimental study by substituting all longitudinal steel bars of shear walls with glass fiber-reinforced polymer (GFRP) bars, and the test results indicated that concrete walls reinforced by GFRP bars displayed stable flexural capacity without strength degradation and recoverable behavior up to allowable drift limits. In addition, significant self-centering capacity could also be obtained in concrete columns reinforced with carbon fiber-reinforced polymer (CFRP) bars or basalt fiber-reinforced plastics (BFRP) bars, as compared with traditional RC columns [22,23,24].

Another innovative alternative to materialize resilient concrete components involves the use of weakly bonded ultra-high strength (WBUHS) rebars with spiral grooves on their surfaces, the key aspect of which lies in their high yield strength (1275 N/mm^2^) as well as low bond strength (3.0–4.0 N/mm^2^), relative to normal strength (NS) rebars [25]. The former provided the RC components with high lateral resistance, while the latter could prevent the premature yielding of WBUHS rebars from endowing both drift-hardening capacity and self-centering capacity. Experimental research has confirmed the feasibility of WBUHS rebars in providing resilience for both square and circular RC columns, regardless of whether they were subjected to high axial compression or deformed in double curvature [26,27,28,29]. In addition, more pronounced resilience can be expected when concrete columns are confined by bolted steel tubes, especially those with circular sections [30]. However, in the case of concrete columns with circular cross-sections, the circular arrangement of WBUHS rebars may potentially interfere with the main reinforcing bars of beams at the column-to-beam joints, indicating that it is worthwhile to squarely arrange WBUHS rebars for circular columns with the view of facilitating the construction of beam-column connections.

In the meantime, there is a growing trend towards increased adoption of prefabricated and modular buildings, primarily motivated by the desire to enhance construction quality and reduce construction timelines. Consequently, it is of great significance to materialize the prefabrication of resilient structures reinforced with WBUHS rebars. Moreover, previous research has emphasized that achieving the optimal mechanical properties of UHS rebars requires secure anchoring with sufficient anchoring length at the ends [31,32,33]. To promote the application of WBUHS rebars in precast construction, it is crucial to assess the difference in seismic performance between precast and cast-in-place construction and to determine the required embedment length for WBUHS rebars to achieve their optimal performance.

Based on the aforementioned background, the primary objectives of this research are: (1) to verify the resilience (referred to as drift-hardening capacity and self-centering capacity) of both cast-in-place and precast columns, and (2) to evaluate the seismic behavior of test columns using an analytical method capable of considering the slippage of WBUHS rebars. To this end, a total of eight concrete columns were fabricated and tested under reversed cyclic lateral loading, with experimental variables including reinforcement types (NS rebar or WBUHS rebar), axial load ratios (0.20 or 0.33), construction methods (cast-in-place or prefabrication), and shear span ratios (1.7 or 2.5). Of them, two ductile concrete columns were reinforced with NS rebars and confined by bolted steel tubes to serve as reference specimens, while the other six concrete columns were reinforced with WBUHS rebars and confined by bolted steel tubes to enhance their resilience. The test results were discussed in terms of hysteretic behavior, residual deformation, strain of reinforcements, equivalent viscous damping coefficient (EVDC) and axial deformation. Furthermore, a numerical analysis was performed to evaluate the hysteresis performance of test columns, and the comparison between analytical and experimental results was discussed.

## 2. Experimental Program

### 2.1. Details of Test Columns

**Figure 1:**
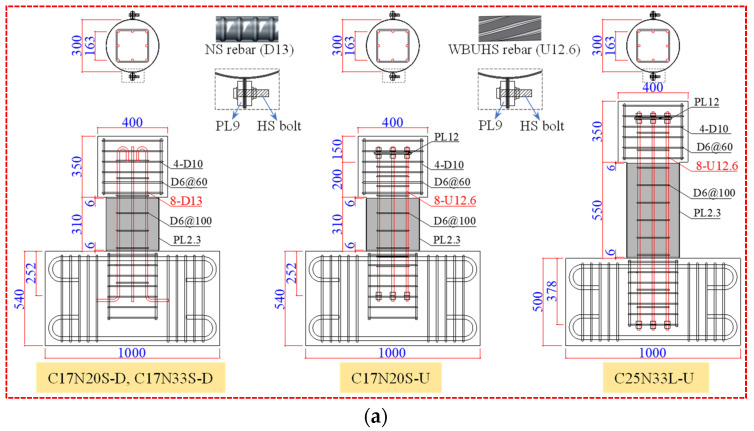

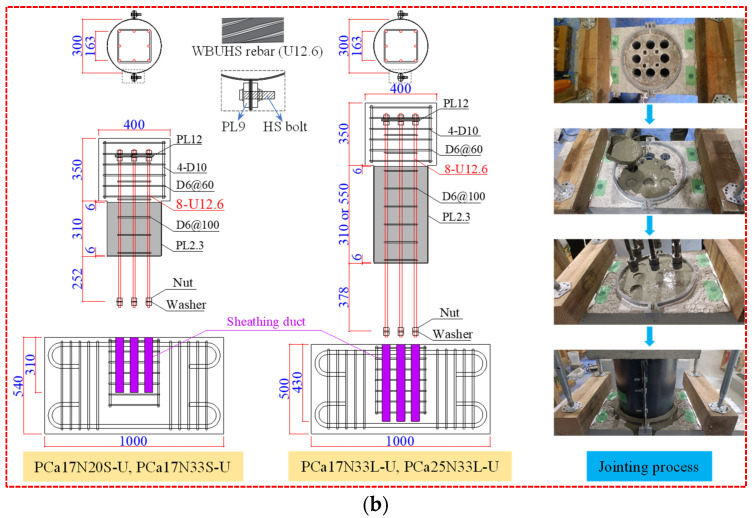
Reinforcement details (unit: mm): (**a**) Cast-in-place columns; (**b**) Precast columns.

A total of eight concrete columns were designed, each composed of a loading stub of 400 × 350 × 350 mm, a circular column with a diameter of 300 mm, and a footing of 1000 × 540 × 400 mm (or 1000 × 500 × 400 mm for the specimens with a shear span ratio of 2.5). Both NS rebars and WBUHS rebars were squarely arranged and transversely confined by D6 deformed rebars with a hoop spacing of 100 mm, meeting the requirements of the hoop reinforcement ratio specified in the AIJ code [34]. For NS rebars, the ends were bent at a 90-degree angle or 180-degree angle to meet anchoring requirements. As for WBUHS rebars, the upper end was secured by high-strength (HS) nuts and a steel plate to ensure the reinforcement position and the lower end was anchored using HS nuts and washers (with outer diameter of 32 mm) to facilitate prefabricated construction. The parameters of test columns are listed in Table 1, and the reinforcement details are depicted in Figure 1.

For the purpose of preventing the early crushing of cover concrete and pursuing significant resilience up to large drift, all the specimens were externally confined by steel tubes with a thickness of 2.3 mm (PL2.3), to give a volumetric ratio of 3.1%. The steel tubes were fabricated by joining two premanufactured semicircular plates through HS bolts and nuts, and steel plates with 9 mm thickness (PL9) were added to prevent the local buckling of the flange (see Figure 1). To prevent the bolted steel tubes from directly carrying axial load, a clearance of 6 mm was maintained between the steel tubes and the loading stub (or the footing). For precast columns, the column and footing were manufactured and poured separately. After one week of pouring, the concrete strength could meet the requirements of removing the formwork and lifting operation. Then, WBUHS rebars protruding from the column were inserted into sheathing ducts pre-embedded in the footing and jointed using high-strength and no-shrinkage grouting material. The jointing process of precast columns was described in Figure 1b.

### 2.2. Mechanical Properties of Materials

The specimens were cast using ready-mixed concrete with a nominal strength of 27 N/mm^2^ and a maximum coarse aggregate particle size of 20 mm, which is commonly employed for constructing the main supporting members. On the basis of the test performed on three cylinders (100 mm in diameter and 200 mm in height), the actual compressive strength (*f_c_′*) of the concrete for each loading day is presented in Table 1. The mechanical properties of utilized materials are given in Table 2.

### 2.3. Test Apparatus and Loading Program

**Figure 2:**
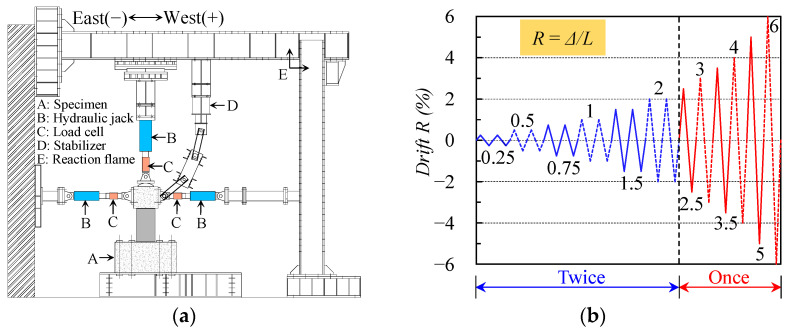
Test apparatus and loading program: (**a**) Test apparatus; (**b**) Loading program.

The test apparatus and loading program of the test columns are illustrated in Figure 2. First, the footing was secured to the strong floor by eight high-strength steel bars with a diameter of 21 mm. Next, a constant axial load (calculated according to the axial load ratio) was applied to the top surface of the loading stub via a vertical jack with 1000 kN capacity. Finally, reversed cyclic lateral loading was imposed using two lateral jacks with a maximum capacity of 500 kN and controlled by the drift *R*, calculated by dividing the lateral displacement (Δ) at the loading point by the shear span (*a*). Two complete cycles were applied within 2% drift, followed by one cycle at subsequent drifts of 2.5%, 3%, 3.5%, 4%, 5% and 6%.

### 2.4. Instrumentation

**Figure 3:**
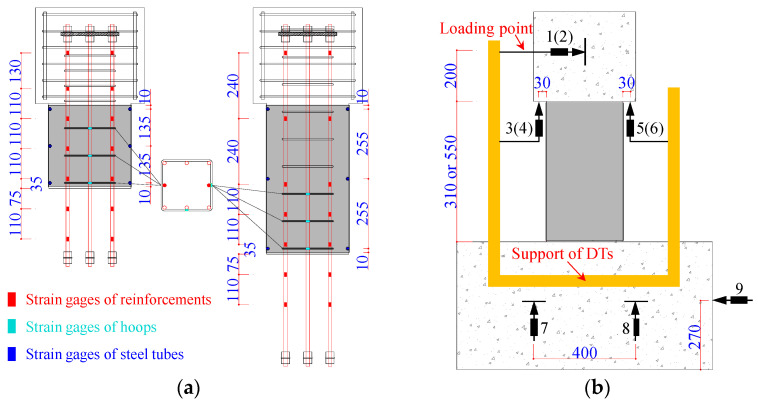
Locations (unit: mm): (**a**) Strain gages; (**b**) Displacement transducers.

The location of displacement transducers (DTs) and strain gages is displayed as Figure 3. The support of DTs was tightly fixed to the footing to form a rigid whole. Two lateral DTs (1−2) were installed in the loading stub to measure the lateral displacement (*Δ*) at the loading point and four vertical DTs (3–6) were placed to measure the axial deformation between the loading stub and footing. DTs (7–9) were used to monitor the displacement variation of the footing in real time during the loading process. Strain gages were attached to NS rebars, WBUHS rebars and steel tubes to serve the subsequent analysis of their mechanical properties.

## 3. Experimental Results and Discussion

### 3.1. Failure Mode

**Figure 4:**
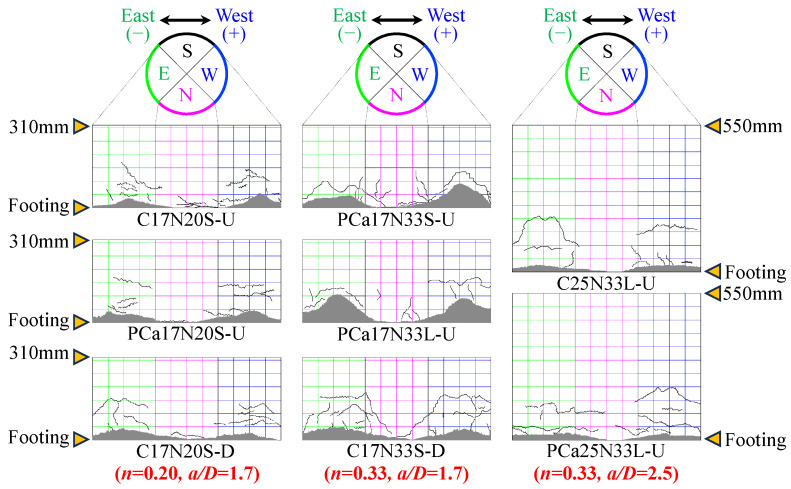
Damage condition of the specimens.

Due to the confinement of steel tubes, it was challenging to observe the damage condition of the specimens during the loading process. Figure 4 illustrates the damage condition of the columns after removing the steel tubes following the completion of loading, with the gray shaded areas representing crushed concrete. The yielding of steel tubes was monitored prior to the specimens reaching the maximum lateral load. Pullout of the WBUHS rebars or pre-embedded sheathing ducts was not observed, indicative of the reliability of the anchoring length for WBUHS rebars and the jointing method for the precast columns. Besides, concrete damage in all specimens was mainly concentrated in the height range of 25 mm to 90 mm at the bottom of the columns with no obvious principal shear fracture or sudden decrease in bearing capacity occurring, implying that the failure mode of all the specimens was dominated by bending. As the axial load ratio increased, the damage to concrete on the compression side of test columns with *a*/*D* = 1.7 became more pronounced and the corresponding range also expanded. Because a larger axial load ratio results in a higher stress level for the concrete in compressive side [35,36].

### 3.2. Lateral Force Versus Drift Relationship

**Figure 5:**
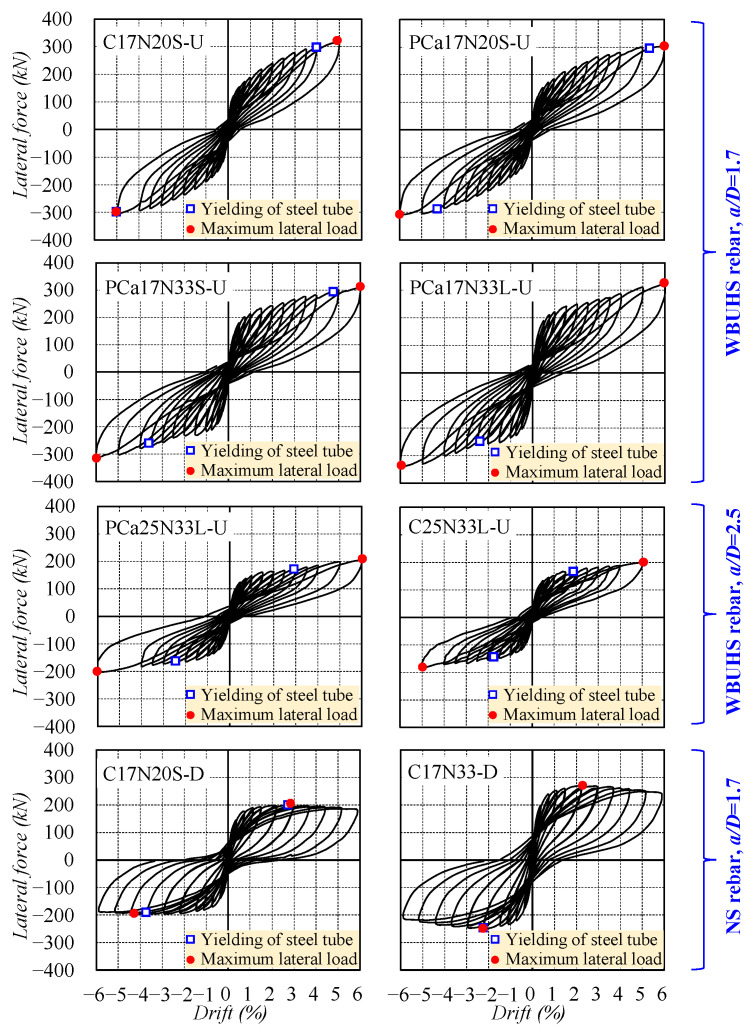
Lateral force versus drift relationship.

The measured lateral force versus drift relationships of the specimens are illustrated in Figure 5, in which the blue blocks and red dots represent the yielding of steel tubes in the transverse direction and the maximum lateral load, respectively. It is noted that drifts in the two ductile concrete columns (C17N20S-D and C17N33S-D) are not completely consistent with the scheduled loading program shown in Figure 2b.

The six concrete columns reinforced with WBUHS rebars exhibited ever-increasing lateral resistance without strength degradation but slow-growing residual deformation even up to a large drift of 5% (surpassing the seismic drift limitations specified in most building codes [37,38]), implying significant drift-hardening capacity and good self-centering capacity even when struck by unexpected severe or mega earthquakes. It can be inferred that the stable lateral response after yielding of steel tubes mainly depends on WBUHS rebars since the yielding of steel tubes usually signifies a maximum confinement effect on the concrete. While the two ductile concrete columns reinforced with NS rebars reached their ultimate lateral loads within 3% drift (see Table 1), the cyclic behavior then began to decrease but with limited strength degradation (5.6% for C17N20S-D and 12% for C17N33S-D at 6% drift) owing to the confinement of the steel tubes, indicating favorable load-holding capacity. Moreover, traditional “fat” hysteresis loops were also observed in the two ductile columns, showing higher absorbed energy but accompanied by an obvious increase in residual deformation.

**Figure 6:**
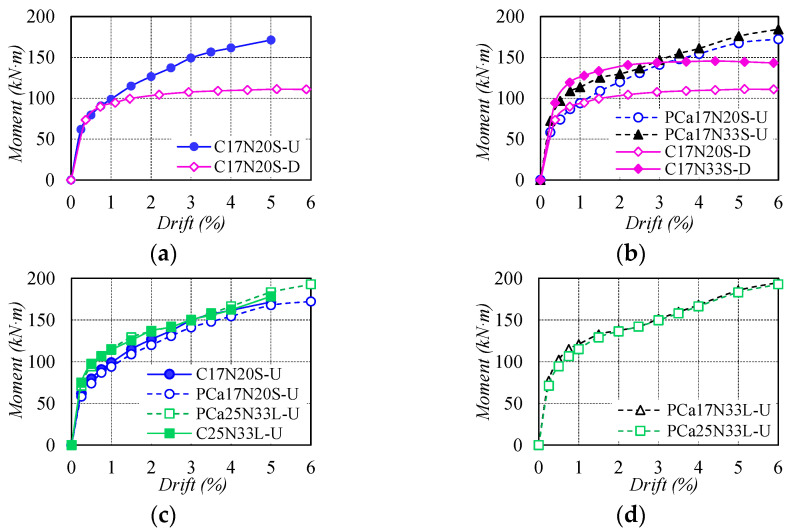
Comparison of envelope curves of bending moment-drift relationships: (**a**) Types of longitudinal rebars; (**b**) Axial load ratio; (**c**) Construction method; (**d**) Shear span ratio.

Figure 6 illustrates the comparison of the envelope curves of bending moment–drift relationships obtained by averaging positive and negative directions. In Figure 6a, the test columns reinforced with NS rebars or WBUHS rebars displayed almost the same moment resistance at the incipient drift (within 0.5%). After the premature yielding of NS rebar at 0.75% drift, the former exhibited slow-growing moment resistance in the succeeding cycles, while the latter performed a remarkable increase in moment resistance due to the high yield strength of WBUHS rebars. As depicted in Figure 6b, the columns with *n* = 0.33 showed higher moment resistance, as the higher axial compression consistently led to a larger neutral axis depth or depth of compression zone. Figure 6c indicated that the precast columns could exert nearly identical moment resistance when the embedment length of the WBUHS rebar was 20 times its diameter. Besides, the moment resistance appears to remain less affected by shear span ratios, as seen in the comparison between PCa17N33L-U and PCa25N33L-U in Figure 6d.

### 3.3. Self-Centering Capacity

**Figure 7:**
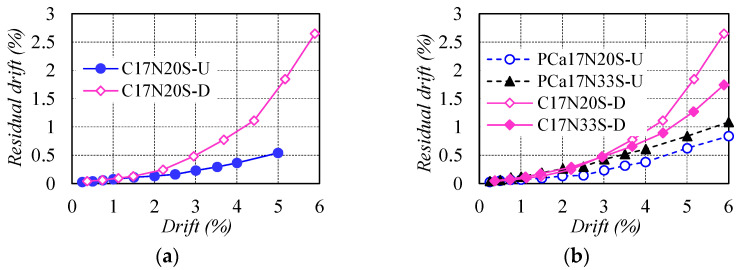

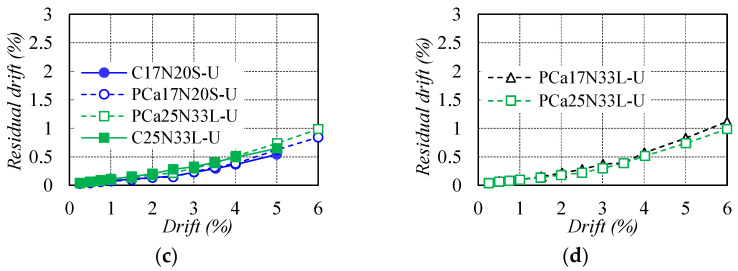
Comparison of residual drift: (**a**) Type of longitudinal rebars; (**b**) Axial load ratio; (**c**) Construction method; (**d**) Shear span ratio.

The residual drift was compared in Figure 7 to evaluate the self-centering capacity of test columns. It can be seen in Figure 7a that the difference in residual drift between C17N20S-D and C17N20S-U was not apparent before the drift of 0.75%. Subsequently, conspicuously increased residual drift was observed in the former due to the yielding of NS rebars, while the residual drift for the latter was still controlled at a low level of no more than 0.54% and even deformed to 5% drift, which was only about one-third of that of C17N20S-D, suggesting remarkable self-centering capacity. For the columns reinforced with WBUHS rebars, the higher the axial compression, the larger the residual drift (see Figure 7b). Additionally, the specimens fabricated with different construction methods or shear span ratios exhibited comparable self-centering capacity as represented in Figure 7c,d.

**Figure 8:**
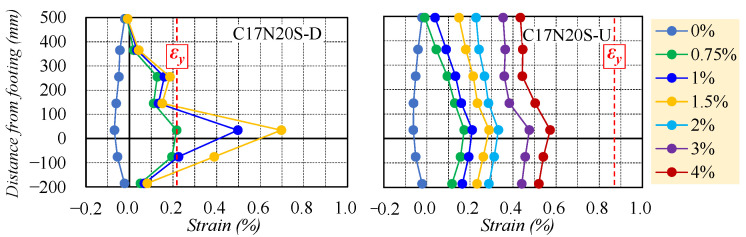
Strain distribution of reinforcements.

Since the self-centering capacity was closely related to the behavior of reinforcements, the strain distribution of reinforcements along the column height at several controlling drifts was plotted in Figure 8 to determine the effect of reinforcement types on the residual deformation observed in Figure 7a. The horizontal black chain dotted line and vertical red dotted line in Figure 8 represented the anchorage positions and yielding of reinforcements, respectively. Results for C17N20S-D are not plotted from the drift of 2.0% because of the sudden failure of partial strain gages located in the plastic hinge region, which possibly resulted from the large plastic deformation of the NS rebars after yielding.

As drawn in Figure 8, the NS rebar in C17N20S-D reached the yield strain at 0.75% drift, followed by a sharp increase in strain observed near the critical section (35 mm from the top of the footing). In contrast, the WBUHS rebar along the column height in C17N20S-U displayed a very small strain gradient. This phenomenon is attributed to the lower bond strength of WBUHS rebars, which facilitates strain penetration along the column height toward the anchorage positions, thereby mitigating strain concentration in the potential plastic hinge region. As a result, this mechanism delays the premature yielding of WBUHS rebars and contributes to an effective self-centering capacity.

### 3.4. Energy Dissipation Capacity

**Figure 9:**
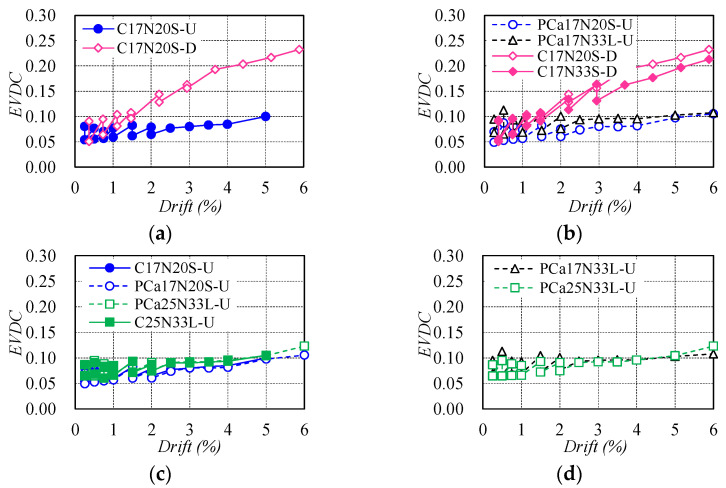
Comparison of equivalent viscous damping coefficient (EVDC): (**a**) Type of longitudinal rebars; (**b**) Axial load ratio; (**c**) Construction method; (**d**) Shear span ratio.

As a critical index for evaluating the energy dissipation of engineering components, equivalent viscous damping coefficient (EVDC) of test columns was calculated following the method proposed by Chopra [39] and was compared in Figure 9. As is obvious in Figure 9a,b, the EVDC in ductile concrete columns increased rapidly owing to the inelastic deformation after yielding of NS rebars. However, in the case of the resilient columns reinforced with WBUHS rebars, the EVDC tended to approach a constant value with the increasing of the drift (see Figure 9b–d), which agrees well with the findings in the study conducted by Sun et al. [30]. This suggests that the columns reinforced with WBUHS rebars exhibited nonlinear elastic behavior even when deformed to a large drift.

### 3.5. Axial Strain

**Figure 10:**
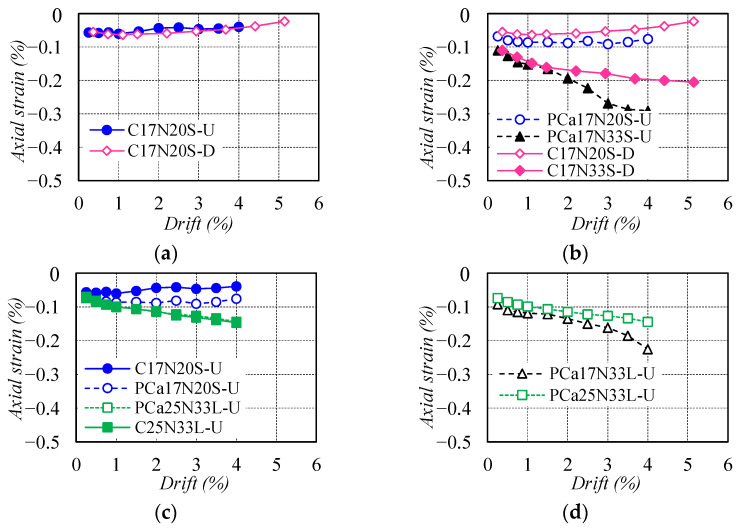
Comparison of axial strain between the footing and loading stub: (**a**) Types of longitudinal rebars; (**b**) Axial load ratio; (**c**) Construction method; (**d**) Shear span ratio.

The axial deformation between the footing and loading stub was measured via the average of the readings from four vertical DTs (3–6) (see Figure 3b) when each cycle was unloaded to the drift of 0%, and the corresponding axial strain was described in Figure 10, obtained by calculating the ratio of measured axial deformation to the distance (310 mm or 550 mm) between the footing and loading stub. It is evident that the axial strain for columns with *n* = 0.20 generally leveled off as drift increased. The columns with *n* = 0.33 showed increasing axial strain because an increase in the axial load will impose more compressive stresses on the column cross-section. However, the maximum axial strain was restricted to within 0.3% even when deformed to a drift of 4%, far below the peak strain of confined concrete. This suggests that the confinement effect provided by steel tubes can provide the resilient columns good axial stability at large drifts.

## 4. Numerical Analysis of Hysteresis Performance

### 4.1. Procedures of Numerical Analysis

According to the fact stated in Section 3.3 that the WBUHS rebars were far from reaching the yield strain even at a drift as large as 4% (see Figure 8), the traditional analysis methods ignoring the slippage of reinforcements will probably not provide an accurate prediction of the hysteretic performance of the resilient columns reinforced with WBUHS rebars, and a feasible analysis method appropriate for resilient columns was urgently needed. In light of these concerns, this paper adopted the fiber spring element (FSE) method developed by Sun et al. [40] to evaluate the hysteretic performance of WBUHS rebar-reinforced columns, which could take into account both the bond–slip behavior of WBUHS rebars and the confinement effect of hoops or/and steel tubes.

**Figure 11:**
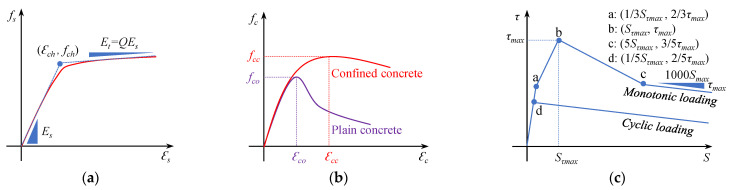
The model utilized in the FSE method: (**a**) UHS rebar; (**b**) Concrete; (**c**) Bond slip.

In this method, the following basic assumptions are made: (1) only WBUHS rebars resist tensile stress; (2) only the concrete part of the section remains plane after bending; (3) the constitutive laws of WBUHS rebars and concrete, respectively, follow the models proposed by Menegotto and Pinto [41] (see Figure 11a), and Sun and Sakino [42] (see Figure 11b); (4) the bond–slip behavior of WBUHS rebars follows the model developed by Funato et al. [25] (see Figure 11c); and (5) the lateral deformation of test columns concentrates in the plastic hinge region with a length of 1.0D (D is the diameter of section) [25], within which the strain and stress of the WBUHS rebars are distributed uniformly.

It is worth highlighting that the strength-raising coefficient (*K*) calculated based on the Sakino–Sun model (derived from welded steel tubes) [42] tends to overestimate the strength of concrete confined by bolted steel tubes because of the discontinuity. This was also supported by the axial compression test results of short concrete columns confined by steel tubes, formed by the same joining method as described in this paper, and 0.49 times the calculated *K* value based on the Sakino–Sun model was adopted for the subsequent numerical analysis [43].

**Figure 12:**
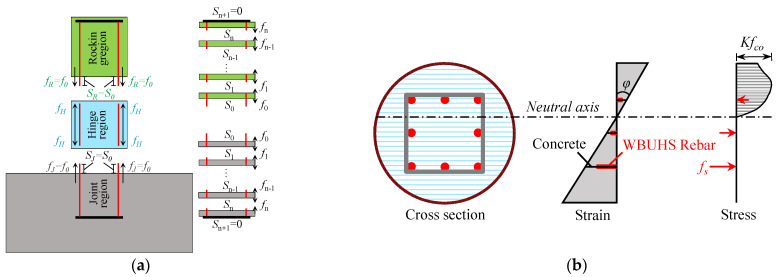
The schematic diagram: (**a**) section division; (**b**) strain distribution.

**Figure 13:**
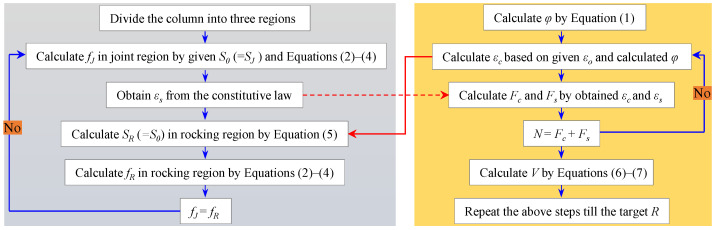
The detailed flow chart of the FSE method.

The schematic diagram of section division and strain distribution are depicted in Figure 12a and Figure 12b, respectively. The detailed flow chart of the FSE method is presented in Figure 13, and the analysis procedure is summarized in the following steps.

1. Divide the column into three regions and discretize the joint and rocking region equally into tiny segments (see Figure 12a).
2. Give an initial concrete strain *ε_o_* at the center of section, and obtain the strain distribution of concrete *ε_c_* along the depth of section (see Figure 12b) based on Equation (1).

(1) $$\varphi =\frac{R}{l_{h}\cdot \left(1-l_{h}/2a\right)}$$

where *φ* is the sectional curvature in the hinge region; *R* is the drift ratio; *l_h_* is the plastic hinge length; and *a* is the shear span.

3. Give an initial stress *f_0_* (=*f_J_*) and slip *S_0_* (=*S_J_*) of the WBUHS rebar in the first segment of the joint region. According to the relationships between the stress (*f_k+_*_1_) and slip (*S_k+_*_1_) listed by Equations (2) and (3), it is necessary to judge whether the boundary condition (see Equation (4)) is satisfied, if not, regive a *f_0_* and *S_0_* and repeat this process until S*_n_*_+1_ = 0 is satisfied. Then the strain of WBUHS rebar *ε_s_* can be obtained from the constitutive law.

(2) $$f_{k+1}=f_{k}-\tau_{k}\cdot \frac{4l}{d}$$

(3) $$S_{k+1}=S_{k}-\varepsilon_{k+1}\cdot l$$

(4) $$S_{n+1}=0$$

where *f_k_* and *f_k+_*_1_ represent the stress of the WBUHS rebar in the *k-th* segment and *k +* 1*-th* segment, respectively; *S_k_*, *S_k+_*_1_ and S*_n+_*_1_ represent the slip of the WBUHS rebar in the *k-th*, *k +* 1*-th* and *n +* 1*-th* segment, respectively; *τ_k_* is the bond stress in the *k-th* segment; *l* represents the length of each segment; and *d* is the diameter of WBUHS rebar.

4. Calculate the slip *S_0_* (=*S_R_*) of the WBUHS rebar in the first segment of the rocking region based on Equation (5). The stress *f_0_* (=*f_R_*) of the rebar in the first segment of the rocking region can be computed by the same procedure shown in step (3).

(5) $$S_{R}=\left(\varepsilon_{c}-\varepsilon_{s}\right)\cdot l_{h}-S_{J}$$

where *S_R_* and *S_J_* represent the slip of the WBUHS rebar in the rocking and jointing region, respectively; and *ε_s_* and *ε_c_* represent the strain of concrete and WBUHS rebars in the hinge region, respectively.

5. Obtain the target value of strain and stress of the WBUHS rebar assumed in step (3) by repeating steps (3)–(4) until *f_J_* = *f_R_* was satisfied.
6. Calculate the force *F_c_* and *F_s_* borne by concrete and WBUHS rebars.
7. Verify whether *N* = *F_c_* + *F_s_* was satisfied. If not, reassign a new center strain *ε_o_* and repeat the process from step (2).
8. Calculate lateral force *V* based on Equations (6) and (7).

(6) $$M=\sum f_{s}A_{s}h_{s}+\sum f_{c}A_{c}h_{c}$$

(7) $$V=\frac{M}{a}-N\cdot R$$

where *f_s_* and *f_c_* represent the stress of WBUHS rebars and concrete, respectively; *A_s_* and *A_c_* represent the area of WBUHS rebars and concrete, respectively; *h_s_* and *h_c_* represent the distance from the acting point of force borne by WBUHS rebars and concrete to the neutral axis, respectively; *N* represents the applied axial force.

9. Repeat the above steps until the target *R*.

### 4.2. Discussion of Analytical Results

**Figure 14:**
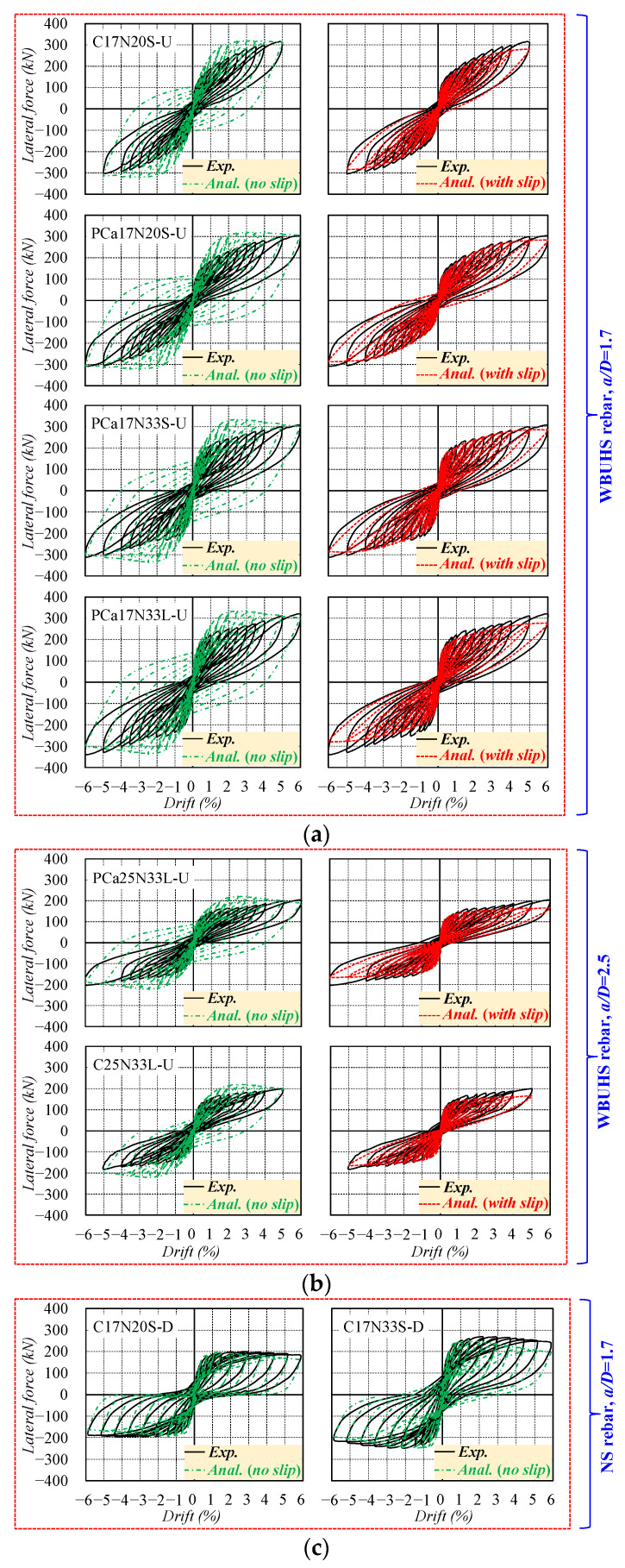
Comparison of the hysteretic performance: (**a**) Concrete columns with WBUHS rebars and *a/D* = 1.7; (**b**) Concrete columns with WBUHS rebars and *a/D* = 2.5; (**c**) Concrete columns with NS rebars and *a/D* = 1.7.

**Figure 15:**
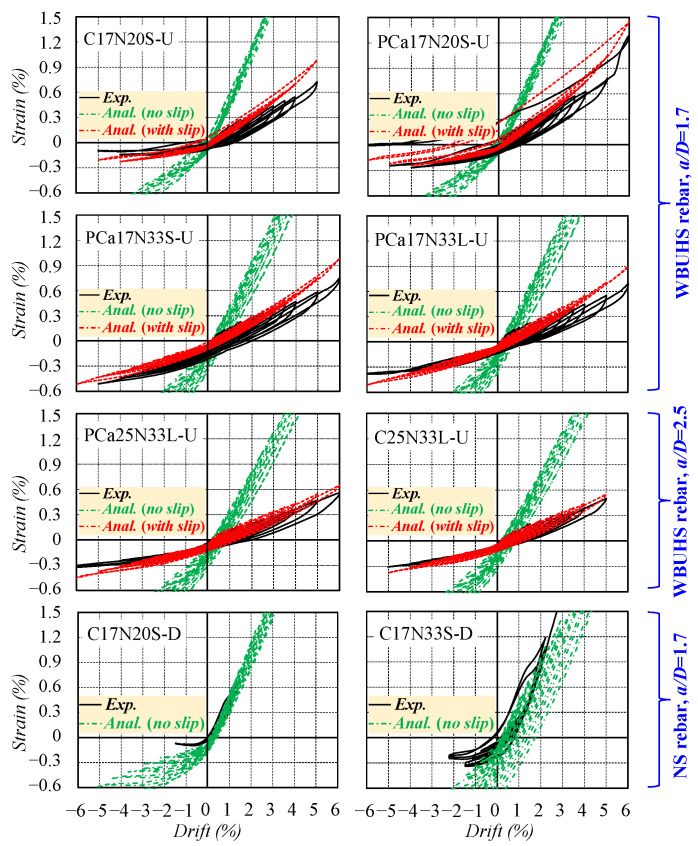
Comparison of reinforcement strain.

Figure 14 and Figure 15 present a comparison of hysteretic performance and reinforcement strain between the experimental and analytical results, respectively. The experimental results were denoted as “*Exp.*”, and the analytical results include two cases: one with consideration for the slippage of WBUHS rebars (denoted as “*Anal. (with slip)*”) and one without (denoted as “*Anal. (no slip)*”). On the one hand, the traditional analysis method, neglecting the bond–slip behavior of reinforcements (“*Anal. (no slip)*”), can effectively track the hysteretic performance of the two ductile concrete columns. However, it not only seems to overestimate the residual deformation and energy dissipation capacity but also results in an overestimation of the lateral resistance of the concrete columns reinforced with WBUHS rebars by 26.9–37.3%. On the other hand, the analysis results with consideration for the slippage of reinforcements (“*Anal. (with slip)*”) is capable of capturing the hysteretic performance of the test columns reinforced with WBUHS rebars, characterized by both remarkable drift-hardening capacity and an evident pinching effect.

The phenomenon described above can be further explained by the comparison of reinforcement strain presented in Figure 15. The analysis result “*Anal. (no slip)*” exhibits good agreement with the experimental results of the NS rebars strain, but the strain of WBUHS rebars increases at a rapid rate and reaches the yield strain at a drift lower than 2%, which inevitably leads to an overestimation of the lateral resistance at the early cyclic loads as compared with experimental results. The analysis result taking into account the slippage of reinforcements (“*Anal. (with slip)*”) can well predict the strain development trend of WBUHS rebars, indicating the validity and accuracy of the FSE method adopted in this paper.

## 5. Conclusions

This paper investigated the seismic behavior of circular concrete columns reinforced with WBUHS rebars and confined by bolted steel tubes. Eight concrete columns were fabricated and tested under reversed cyclic lateral loading simultaneously subjected to constant axial compression. Based on the experimental and analytical results, the following conclusions can be drawn.

- As compared with traditional ductile concrete columns, the concrete columns reinforced with WBUHS rebars exhibited significant drift-hardening capacity as well as self-centering capacity up to the drift of at least 5%.
- If the embedment length of the WBUHS rebar was 20 times its diameter, the precast concrete columns could exert almost the same excellent seismic performance as cast-in-place columns.
- The test columns under higher axial compression showed comparatively serious concrete damage, but larger moment resistance.
- The analytical results, considering the slippage of reinforcements, effectively predicted the strain development trend of WBUHS rebars, and demonstrated good agreement with the hysteretic performance of test columns reinforced with WBUHS rebars.
- In light of the significant drift-hardening capacity and good self-centering capacity exhibited by the concrete columns reinforced with WBUHS rebars and confined by steel tubes. The use of resilient concrete columns developed in this paper is applicable to the ground floors of mid- and high-rise buildings to reduce the need for maintenance and repairs, particularly for structures in high seismic-intensity areas.

## Figures and Tables

**Table 1:** Parameters and measured peak load of the test columns.

Specimen	Longitudinal Rebar	*a/D*	Construction Method	*l_e_*	*n*	Lateral Confinement	*f_c_′* (MPa)	*Q_exp_* (kN)	*R_exp_* (%)
C17N20S-U	8-U12.6	1.7	Cast-in-place	20d	0.20	D6@100 + PL2.3	38.1	309.7	5.0
PCa17N20S-U	8-U12.6	1.7	Prefabrication	20d	0.20	D6@100 + PL2.3	38.5	306.0	6.0
PCa17N33S-U	8-U12.6	1.7	Prefabrication	20d	0.33	D6@100 + PL2.3	38.4	309.8	6.0
PCa17N33L-U	8-U12.6	1.7	Prefabrication	30d	0.33	D6@100 + PL2.3	38.0	330.1	6.0
PCa25N33L-U	8-U12.6	2.5	Prefabrication	30d	0.33	D6@100 + PL2.3	41.3	190.1	6.0
C25N33L-U	8-U12.6	2.5	Cast-in-place	30d	0.33	D6@100 + PL2.3	40.6	199.1	5.0
C17N20S-D	8-D13	1.7	Cast-in-place	20d	0.20	D6@100 + PL2.3	41.7	193.6	3.6
C17N33S-D	8-D13	1.7	Cast-in-place	20d	0.33	D6@100 + PL2.3	42.8	254.5	2.2

Note: *a/D*: shear span ratio, *l_e_*: embedment length of longitudinal rebar, *n*: axial load ratio, *f_c_′*: the compressive strength of concrete at each loading day, *Q_exp_*: the average value of measured peak load in positive and negative directions, *R_exp_*: drift at peak load.

**Table 2:** Mechanical properties of utilized materials.

Name	Diameter or Thickness (mm)	Yield Strength (N/mm^2^)	Yield Strain (%)	Tensile Strength (N/mm^2^)	Elasticity Modulus (kN/mm^2^)
WBUHS rebar (U12.6)	12.6	1401	0.86	1478	212
NS rebar (D13)	12.7	403	0.22	600	200
Hoop (D6)	6.35	400	0.22	525	197
Steel tube (PL2.3)	2.3	391	0.20	465	201

## Data Availability

The data that support the findings of this study are not publicly available at this time as the data also forms part of an ongoing study but are available from the corresponding author on reasonable request.
